# Can Self-Esteem Help Teens Resist Unhealthy Influence of Materialistic Goals Promoted By Role Models?

**DOI:** 10.3389/fpsyg.2021.687388

**Published:** 2022-01-04

**Authors:** Anna Maria Zawadzka, Judyta Borchet, Magdalena Iwanowska, Aleksandra Lewandowska-Walter

**Affiliations:** Faculty of Social Sciences, Institute of Psychology, University of Gdańsk, Gdańsk, Poland

**Keywords:** materialism, self-esteem, role models, parents, peers, media

## Abstract

The aim of the study was to examine the role of self-esteem in resisting the influence of materialistic goals of four social role models (mother, father, peers, and media) in adolescents (aged 13–16). Previous studies showed a negative correlation between the psychological health of teens and striving for materialistic goals, one of the main sources is the social modeling of materialism. Two studies were carried out. The first, correlational study, was conducted on target teens and their mothers, fathers, and peers of their choice. It examined if self-esteem is a moderator of the relationship between the materialism of social role models (mothers, fathers, peers, and media) and the materialism of teens. The second, experimental study, was conducted on target teens only. It examined how boosting the self-esteem of teens and activating materialism of social role models (mothers, fathers, peers, and media) may affect the materialism of teens. Study 1 showed a significant interaction effect of self-esteem and the materialism of peers on the materialism of teens. The interaction effects of self-esteem and other role models (parents and media) were not significant. Study 2 showed that elevated self-esteem lowered the influence of the materialism of peers on the materialism of teens. The results were not significant when other role models (parents and media) were analyzed. The results obtained in the presented studies indicate that the self-esteem of teens may have an important role in resisting the influence of materialism role models of peers. Practical implications of the studies for the psychological health of teens are also discussed.

## Introduction

Self-determination theory holds that the importance attached to the pursuit of intrinsic and extrinsic goals is related to the wellbeing of an individual (Ryan and Deci, 2000). Intrinsic goals, i.e., self-acceptance, affiliation, and community feeling, satisfy basic psychological needs, and striving for these is in itself rewarding. Extrinsic goals, i.e., financial success, popularity, and image (physical appearance), also known as materialistic goals, refer to focusing on external rewards, opinions of other people, and making an impression on others. Self-determination theory (SDT) assumes that the pursuit of extrinsic goals is problematic only when they are considered more important than intrinsic goals. In line with this approach, in this paper, materialism is understood as an orientation toward materialistic goals, i.e., financial success, popularity, and physical appearance, at the cost of neglecting non-materialistic goals, i.e., self-acceptance, affiliation, and community feeling; (Relative Extrinsic Versus Intrinsic Value Orientation, REIVO) (Kasser, 2002; also see Kasser et al., 2014).

Psychological research confirms that successive generations of teens are becoming more materialistic (Twenge and Kasser, 2013). And yet, existing literature demonstrates that attaching excessive significance to materialistic goals and attaching greater significance to materialistic goals than intrinsic goals may cause negative consequences for physical health, psychological condition, and social wellbeing of adolescents (Kasser and Ryan, 1993; Cohen and Cohen, 1996; Williams et al., 2000; Vansteenkiste et al., 2007; Dittmar et al., 2008; Twenge et al., 2010; Kasser et al., 2014; Tsang et al., 2014; Moldes and Ku, 2020).

One of the main sources of the materialism of teens is social modeling, and there are various role models (parents, peers, and media) that can shape the materialism of teens (Kasser et al., 1995; Flouri, 1999; Goldberg et al., 2003; Chaplin and John, 2010; Zawadzka and Dykalska-Bieck, 2013; Zawadzka et al., 2017, 2021; Chaplin et al., 2019). As a result of materialism social modeling, the individual becomes oriented toward pursuing materialistic goals (Kasser, 2002).

In view of the negative health consequences of materialism for adolescents, it is important to identify ways in which the impact of materialism role models can be lessened. One of the possible resources associated with resistance to the influence of materialism role models is high self-esteem. Self-esteem is a positive attitude toward oneself. It is an effective resource, which brings a sense of social support (Battistich et al., 1993; Keefe and Berndt, 1996; Glendinning and Inglis, 1999; Leary and Baumeister, 2000) and works as a mechanism that can deal with the problem of being excluded from a group (cf. Achenreiner, 1997; Banerjee and Dittmar, 2008; Jiang et al., 2015). Furthermore, lower self-esteem is associated with pursuing materialistic goals (Chaplin and John, 2007; Park and John, 2011; Kasser et al., 2014; Liang et al., 2016; Zawadzka and Iwanowska, 2016). Thus, individuals with elevated self-esteem seem likely to be more resistant to the social modeling of materialism. The presented study is an attempt to answer the question of whether the self-esteem of teens may be a resource that is associated with resistance to materialism influences.

In this study, we worked toward three goals. First, we analyzed whether elevated teens’ self-esteem can be a moderator of the relationship between the materialism of role models (mothers, fathers, peers, and media) and the materialism of teens. Second, we checked whether boosting self-esteem can lessen the influence of materialism role models (mothers, fathers, peers, and media). Third, we examined whether higher self-esteem may help teens to resist the influence of any of the materialism role models in question. Below, we present the theoretical basis of the study and discuss questions posed in our two studies, correlational and experimental.

### Self-Esteem and Materialism of Teens

Self-esteem can be analyzed as a one-dimensional global self-evaluation (Rosenberg, 1965) and as a multi-dimensional self-evaluation in various domains (Chen et al., 2020). Self-esteem is understood in this paper as positive global self-evaluation and a feeling of general happiness and satisfaction (Harter, 1999). According to self-determination theory, self-esteem occurs as the effect of satisfying basic psychological needs (i.e., affiliation, autonomy, and competence) and pursuing intrinsic, non-materialistic, goals (i.e., self-acceptance, affiliation, and community feeling; Ryan and Deci, 2000; Kasser, 2002). Materialistic goals are in opposition to intrinsic, non-materialistic, goals – the pursuit of the former occurs with neglect of the latter. Studies on teens indicate that materialism can be a compensatory strategy for violation of self-esteem (Chaplin and John, 2007; Park and John, 2011; Shrum et al., 2013; Kasser et al., 2014; Zawadzka and Iwanowska, 2016). This compensatory strategy occurs more frequently in teens from families with low social-economic status (SES) than in those from families with high SES (Nairn and Opree, 2021).

Studies on teens to date mainly deal with the relationship between self-esteem and materialism. They show that lowered self-esteem is linked to increased materialism (Chaplin and John, 2007). Other studies also say that the negative relationship between self-esteem and materialism is stronger for implicit than explicit self-esteem (Park and John, 2011). However, in one study, the negative relationship between self-esteem and materialism was confirmed by an indirect measure and was not confirmed by a direct one (Zawadzka and Iwanowska, 2016). Other studies indicate that the negative relationship between self-esteem and materialism depends on how the teens define themselves (Gil et al., 2016; Zhang and Hawk, 2019; Zhang et al., 2020) and how stable the self-esteem is (Baoyan et al., 2021). Self-esteem also directly affects body image and body satisfaction, which are related to materialism (Guðnadóttir and Garðarsdóttir, 2014; Sun, 2018). Some studies show a causal direction between materialism and self-esteem, e.g., they found that activating positive self-beliefs reduces materialism (Chaplin and John, 2007; Liang et al., 2016). In a longitudinal study by Kasser et al. (2014), decreasing materialism resulted in increasing self-esteem, especially in those participants who had had a high level of materialism.

Thus, most previous studies show that materialism of teens is negatively linked to high/elevated self-esteem and that materialism may be a compensatory strategy for the decline of self-esteem.

### Parents, Peers, and Media as Materialism Role Models

Theories of social modeling postulate that certain behaviors and attitudes arise through observation and imitation of role models – mothers, fathers, peers, and those displayed by media (Bandura, 1997). Many studies to date demonstrate that adolescent materialism is linked to the materialism of role models. Moreover, experimental studies indicate that activation of various materialism role models may increase the importance of materialistic aspirations of teens (Zawadzka et al., 2021).

Cross-sectional studies indicate that the materialism of teens is positively linked to the materialism of mothers (Flouri, 1999; Goldberg et al., 2003; Chaplin and John, 2010; Zawadzka and Dykalska-Bieck, 2013). As far as the link between the materialism of fathers and materialism of teens are concerned, the results are inconsistent; some show significant positive relationships (Goldberg et al., 2003; Chaplin and John, 2010), while others do not show any significant relationships (Flouri, 2004; Wojtowicz, 2013; Zawadzka and Dykalska-Bieck, 2013).

As regards peers, studies show that teens learn materialism from peers and friends (Churchill and Moschis, 1979; Santini et al., 2017), teens who have materialistic peers display higher levels of materialism (Sheldon et al., 2000; Chaplin and John, 2010), peer influence relates to the materialism of teens (Vinayak and Arora, 2018) and more intense involvement in consumption (cf. Bachmann et al., 1993; Gentina and Bonsu, 2013), and materialism of teens is positively linked to susceptibility to peer influence (Achenreiner, 1997).

Research into media and materialism of teens shows that media play a role in increasing the materialism of children and teens (Goldberg and Gorn, 1978; Churchill and Moschis, 1979; Moschis and Moore, 1982; Buijzen and Valkenburg, 2003; Schor, 2004; Chia, 2010; Sharif and Khanekharab, 2017; Wang et al., 2020). Media messages and advertisements exposure may foster the conviction that money, fame, and attractive image are the source of happiness in life and, in this way, are important life goals. However, correlations between media exposure and adolescent materialism are relatively low.

As described above, the research literature indicates that parents, peers, and media affect the materialism of teens. However, the impact of these role models can be different (cf. Parke, 2004). In adolescence, a period of maturation, teens increasingly crave relations with their peers, who experience similar problems (cf. Steinberg and Morris, 2001). Consequently, peer pressure becomes stronger, and the importance of conforming to group norms increases while parental influence decreases (Schaffer, 1996). Adolescence is also a period when teens are more prone to risky behaviors (Garcia et al., 2020). Peers can have both a positive impact on the teen, e.g., contribute to the development of social competencies (Gallarin and Alonso-Arbiol, 2012) and a negative one, e.g., encourage to undertake unhealthy and risky behaviors (Ridao et al., 2021). Although parental influence decreases during adolescence decreases, it is still important for the optimal functioning of the teen. Teens who have caring and committed parents tend to have higher self-esteem and adapt better to school and are less likely to engage in unhealthy and risky behaviors than those whose parents are cold and neglectful (Yeung, 2021). The materialism of teens is increased by both the normative influence of peers (Achenreiner, 1997; Chan and Prendergast, 2007, 2008) and social comparisons with peers (Chan and Prendergast, 2007, 2008). Peers have an influence on how teenagers receive and evaluate media information (Steinberg and Monahan, 2007). They use the media and get inspiration for shopping, for example, from there (Ward and Wackman, 1971; Shrum, 1996).

Thus, previous studies show that although there are links between the materialism of teens and materialism of all role models (i.e., mothers, fathers, peers, and media), peers may play a special role in modeling the materialistic goals and attitudes of teens.

### Self-Esteem and Social Modeling of Materialism

Self-esteem is also shaped in the context of social relationships. Parental warmth and commitment, unconditional love, and a sense of security offered by parents lay the foundations for positive self-esteem (Coopersmith, 1967; Harter, 1983; Martinez et al., 2020; Queiroz et al., 2020). It is well documented that parental support is vital for self-esteem (Gecas and Schwalbe, 1986; Parker and Benson, 2004; Chaplin and John, 2010) and that positive parent-child interactions can delay the decline of self-esteem in adolescence (Yang and Schaninger, 2010). Teens fulfill the need for intimacy and closeness in friendly relationships with their peers to a greater extent than younger children do (Berndt, 1989; Berndt and Perry, 1990). Having friendly relationships with peers results in elevated self-esteem, while feelings of rejection by peers lead to a drop in self-esteem (Damon et al., 2006). According to current approaches to the function of self-esteem, high self-esteem may serve as an effective resource, which gives a sense of social support (Leary and Baumeister, 2000). Sociometer Theory (Leary and Baumeister, 2000) holds that self-esteem functions as a barometer relational value of a person – high self-esteem is linked to a sense of being accepted and high relational value while low self-esteem is linked to a sense of exclusion and low relational value. Individuals with high self-esteem can feel a sense of social support (Battistich et al., 1993; Keefe and Berndt, 1996; Glendinning and Inglis, 1999; Leary and Baumeister, 2000) and high self-esteem helps them cope with rejection (Brown, 2004). Studies on teens showed that high self-esteem is associated with perceiving oneself as popular among peers, while low self-esteem is associated with perceiving oneself as socially isolated (cf. Glendinning and Inglis, 1999). In addition, individuals with high self-esteem have a stronger influence on interaction partners and see themselves as having more influence than people with low self-esteem (Cohen, 1959). What is more, the experience of being excluded from a peer group is associated with feeling greater pressure from the group and fosters materialism (Achenreiner, 1997; Banerjee and Dittmar, 2008), and, vice versa, materialism resulting from the experience of peer rejection may decrease when (implicit) self-esteem is elevated (Jiang et al., 2015).

Few previous studies analyzed the relations of having or not having social support with materialism and self-esteem as a mediator of such relations. They showed that self-esteem of teens can be a mediator of the relation between perceived acceptance and support of peers or parents and adolescent materialism – acceptance and support elevate self-esteem and, consequently, lower materialism (Chaplin and John, 2010; Fu et al., 2015; Gentina et al., 2018).

In conclusion, both parental and peer support can elevate self-esteem and lower materialism. However, the importance of parental support and acceptance for positive self-esteem is crucial, while the importance of peer support and acceptance for positive self-esteem may be susceptible to change. High/elevated self-esteem may give a sense of social support and help deal with being rejected or excluded from the peer group.

### The Current Study

The aim of the present studies is to fill in the gaps that exist in the literature on teenage materialism (REIVO) and self-esteem. Until now, the role that self-esteem may play in moderating materialistic social influences on adolescents has not been clearly explained in the literature. Based on various findings, we assumed that self-esteem might play a crucial role in resisting the materialistic influence of peers. First, high self-esteem is an emotional resource, which evokes a sense of social support (cf. Battistich et al., 1993; Keefe and Berndt, 1996; Glendinning and Inglis, 1999; Leary and Baumeister, 2000) and elevates the relational value of a person (cf. Leary and Baumeister, 2000; see also Chaplin and John, 2010; Gentina et al., 2018). Second, self-esteem may play a mediating role in the relationship between social support and materialism (Chaplin and John, 2010; Gentina et al., 2018). Third, individuals with higher self-esteem tend to have a stronger influence on their interaction partners than individuals with lower self-esteem (Cohen, 1959). Fourth, the risk of exclusion from a peer group is an important correlate of materialism, and elevating self-esteem lowers materialism resulting from peer group exclusion (Jiang et al., 2015). Thus, we formulated the first hypothesis H1: Self-esteem will moderate the relation between the materialism of peers and the materialism of teens.

Furthermore, previous studies have not examined the role that self-esteem may play in reducing materialistic influences on adolescents. Taking into account, once again, theories about the function of self-esteem as a sense of being accepted (cf. Leary and Baumeister, 2000), and findings suggesting that elevating self-esteem may lower materialism (cf. Chaplin and John, 2007), and considering that materialism of teens resulting from peer rejection may be lowered when self-esteem is elevated (Jiang et al., 2015), we proposed hypothesis H2: Elevating self-esteem will reduce the influence of materialism of peers on the materialism of teens.

To test the hypotheses, we carried out two studies, a cross-sectional one and an experimental one. We analyzed all materialism role models discussed above (i.e., mothers’, fathers’, peers’, and media), to check the assumed effects in both studies.

## Study 1^1^

### Method

The first, cross-sectional, study tests Hypothesis H1 about self-esteem as a moderator of the relationship between the materialism of peers and materialism of teens. In the study, we checked the moderation effect of self-esteem on the relation between the materialism of teens and materialism of each of the role models examined, i.e., mothers’, fathers’, peers’, and media.

#### Participants

We have surveyed 796 subjects. The sample of target teens consisted of 199 middle school students, aged 13–16 (*M*_age_ = 14.36, SD = 1.07), of whom 53.3% were girls and 46.7% were boys. We also obtained data from 199 of their mothers (*M*_age_ = 41.71, SD = 4.06), 178 of their fathers (*M*_age_ = 43.67, SD = 5.32), and 199 of their peers (of whom 57.3% were girls and 42.7% were boys; *M*_age_ = 14.44, SD = 1.57). None of the mothers and fathers had primary school education, 10.1% of the mothers and 19.6% of the fathers had vocational education, 25.1% of the mothers and 24.1% of the fathers had secondary education, 11.6% of the mothers and 6.5% of the fathers had a Bachelor’s degree, and 52.3% of the mothers and 48.7% of the fathers had a Master’s degree or above. All respondents came from the Pomeranian region of Poland. The families maintained an average standard of living (*M* Family SES = 6.20; SD = 1.37) measured by MacArthur’s Scale of Subjective Social Status (Goodman et al., 2001).

In both studies, we surveyed 13–16-year olds. Teens after the period of early adolescence, in which the self-esteem lowers, may show an increase in materialism (cf. Chaplin and John, 2007). The sample consisted of a similar number of girls and boys; studies indicate that the level of the materialism of teenage boys is generally higher than that of teenage girls (cf. Churchill and Moschis, 1979; Achenreiner, 1997). The teens came from families with (similar) family SES; previous research shows that family SES is related to the self-esteem of teens (cf. Nairn and Opree, 2021). The above selection criteria were applied to standardize the sample and minimize a possible impact on the study of the dependencies between age, gender, and family SES.

#### Procedure and Materials

Prior to the study, consent had been obtained from both the research ethics committee at the University [blinded] and from principals of the schools whose students we surveyed. Each parent had been informed about the study and its goals and had allowed the child to participate in the study. The survey was anonymous. The same procedure was followed in both studies presented in this paper. All questionnaires were administered in the Polish language.

The research was conducted at the schools that target teens attended. The teens, their mothers, fathers, and peers filled out questionnaires in groups of 5–15 people. The peers we surveyed were those indicated by target teens as their best friends. The questionnaires consisted of questions about demographics and measures of materialism (refer below). The sets for target teens also included a self-esteem scale and measures of exposure to media and the Internet. The scales and measures were arranged in a random sequence.

##### The Measure of Target Materialism of Teens

We used an adaptation of the Aspiration Index (AI) for teens (AI; Kasser et al., 2014, Study 4). It includes 36 goals grouped in 12 aspiration domains (Affiliation, Self-acceptance, Community, Financial Success, Popularity, Physical Appearance, Hedonism, Safety, Conformity, Spirituality, Health, and Savings). A standard back-Polish-translation of the English version of AI was used (Brislin, 1970). Respondents used a 9-point scale (1 = *not at all important* and 9 = *extremely important*) to rate “How important was each goal to you in the past month.” We wanted to find here how important the three primary extrinsic (materialistic) domains were for the teens, that is a financial success (e.g., *I will have many expensive possessions*), popularity (e.g., *I will be admired by many people*), and physical appearance (e.g., *My image will be one that others find appealing*), relative to the three primary intrinsic (non-materialistic) domains, that is, self-acceptance (e.g., *I will choose what I do, instead of being pushed along by life*), affiliation (e.g., *People will show affection to me, and I will to them*), and community feeling (e.g., *I will assist people who need it, asking nothing in return*). We calculated the level of materialism assuming, in line with the conclusions of Grouzet et al. (2005), that materialistic goals and intrinsic goals are two ends of the same dimension. We summed scores on the three intrinsic domains (nine items) and subtracted them from the sum of scores on the three extrinsic domains (nine items) (cf. Sheldon and Kasser, 1998, Kasser, 2001). In this way, we generated a REIVO score, which reflects the relative importance an individual puts on extrinsic/materialistic vs. intrinsic aspirations (refer to also Kasser et al., 2014). The higher the result, the higher the level of materialism is. The reliability of the REIVO was Cronbach’s α = 0.78 and McDonald’s Ω = 0.80 (refer to Deng and Chan, 2017).

##### The Measure of Target Self-Esteem of Teens

We assessed self-esteem with the Rosenberg Self-Esteem Scale (Rosenberg, 1965; a Polish adaptation by Łaguna et al., 2007) consisting of ten statements relating to feelings about one’s worth. Respondents rated the statements on a 4-point scale (1 = *strongly agree* and 4 = *strongly disagree*). The reliability of the scale was high, Cronbach’s α = 0.86, McDonalds’Ω = 0.87.

##### The Measure of the Materialism of Mothers and Fathers

We used an adaptation of the AI for adults (Kasser and Ryan, 1993; a Polish adaptation by Zawadzka et al., 2015) to assess the materialism of mothers and fathers. It included 35 goals assessing three extrinsic (materialistic) aspirations: financial success, popularity, physical appearance, and three intrinsic (non-materialistic) aspirations: personal growth, affiliation, and community feeling; five items also assessed health aspirations (e.g., to be fit and healthy) but we did not focus on them here. Respondents rated the question of “How important each goal is to you?” using a 7-point scale (1 = *not at all important* and 7 = *very important*) for each item. We created a REIVO score (cf. Kasser et al., 2014). The reliability of the REIVO for mothers was Cronbach’s α = 0.78 and McDonalds’Ω = 0.81 and for fathers was Cronbach’s α = 0.81 and McDonalds’Ω = 0.80.

##### The Measure of the Materialism of Peers

We used the AI for teens described above to assess the materialism of peers. We created a REIVO score (cf. Kasser et al., 2014). The reliability of the REIVO for peers was α = 0.63 and McDonald’s Ω = 0.70.

##### The Measures of Media and Advertising Exposure

We used a method inspired by Schor (2004) by asking target teens to answer questions related to the frequency of their viewing TV and using the Internet. Using a 5-point scale (1 = *never* and 5 = *always*), first respondents indicated how often they watch television and use the Internet on weekdays at five specific times of day (i.e., before school, after school, during dinner, after dinner, and in bed before sleep). Next, using the same 5-point scale, they rated how often they engaged in the same two activities on the weekend at six specific times of day (i.e., in the morning, during lunch, in the afternoon, during dinner, after dinner, and in bed before sleep). We summed the ratings of exposure to television, and the Internet, during the weekdays and weekends.

### Results

#### Preliminary Analysis

Table 1 displays correlations between all studied variables. Target self-esteem of teens was significantly negatively correlated with the materialism of mothers and materialism of peers but was not significantly related to the materialism of teens, the materialism of fathers, and media exposure. Correlations between self-esteem and sex (*r* = − 0.08) and age (*r* = − 0.13) were not significant. The materialism of all role models was positively related to the materialism of teens.

**TABLE 1:** Correlations of variables (Study 1).

Variables	1	2	3	4	5
1. Teenager’s self-esteem					
2. Teenager’s materialism	–0.08				
3. Mother’s materialism	–0.20**	0.32***			
4. Father’s materialism	–0.05	0.31***	0.32***		
5. Peer’s materialism	–0.28**	0.32***	0.32***	0.26***	
6. Media exposure	–0.01	0.14*	0.14*	–0.098	0.07

Level of significance: * = 0.05, ** = 0.01, *** = 0.001.

#### Self-Esteem as a Moderator of the Relationship Between the Materialism of Teens and Materialism Role Models

We examined whether the relationships between the materialism of teens and that of the four role models (e.g., mothers, fathers, peers, and media) were moderated by the self-esteem of teens. Moderation effects were tested with regression analysis using the PROCESS bootstrapping macro procedure (Hayes, 2013).

**FIGURE 1:**
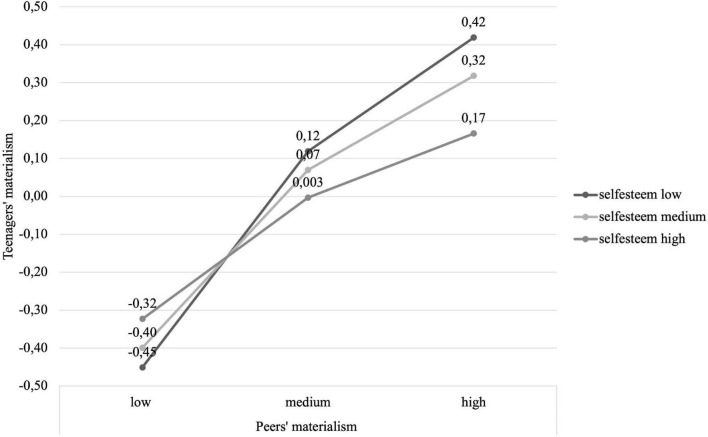
Moderation effect of teenager self-esteem on the relationship between materialism of peer and materialism of teenager (Study 1).

Of the four potential moderation effects of self-esteem, one was significant – self-esteem moderates the relationship between materialism of peers and materialism of teens (Δ*R*^2^ = 0.04, Δ*F*_(1_,_195)_ = 9.69, *b* = − 0.14, SE = 0.04, *t* = − 3.11, *p* = 0.002, lower 95% CI [LLCI] = − 0.222, upper 95% CI [ULCI] = − 0.049). As can be seen in Figure 1, the materialism of teens rises with the increase of materialism of their peers, but the effect is weaker when the self-esteem of teens is high.

The results do not show that self-esteem is a moderator in the case of the other three social materialism role models studied, i.e., mother (Δ*R*^2^ = 0.006, Δ*F*_(1_,_195)_ = 1.19, *b* = 0.08, *t* = 1.09, *p* = 0.276, LLCI = − 0.061, ULCI = 0.213), father (Δ*R*^2^ = 0.006, Δ*F*_(1_,_195)_ = 1.32, *b* = − 0.08, SE = 0.07, *t* = − 0.1.15, *p* = 0.251, LLCI = − 0.225, ULCI = 0.059), and media (Δ*R*^2^ = 0.004, Δ*F*_(1_,_195)_ = 0.735, *b* = − 0.06, SE = 0.07, *t* = − 0.857, *p* = 0.392, LLCI = − 0.201, ULCI = 0.079).

Therefore, the results of Study 1 support hypothesis H1, predicting that the self-esteem is a moderator of the materialism of peers on the materialism of teens.

## Study 2

### Method

The second, experimental study tests Hypothesis H2 relating to the effect of elevating self-esteem on the impact of the materialism of peers on the materialism of teens. In this study, we activated either both self-esteem and materialism of the four role models (mother, father, peer, or media) together or the materialism of the four role models only. Then we tested if elevating the self-esteem of teens can lessen the impact of the materialism of the role models on the materialism of teens as compared to the neutral group (i.e., neither self-esteem was elevated nor materialism role model was activated) as a reference group. In our experimental study, we used semantic priming and goal priming to activate both materialisms of social role models and self-esteem. Exposure to specific stimuli, concepts, or clues concerning certain knowledge or goals makes the knowledge and goals more accessible (cf. Forster et al., 2009). The dependent variable was the materialism of teens measured with AI (i.e., REIVO score) as the relative importance individuals place on extrinsic (materialistic) vs. intrinsic aspirations.

### Participants

The sample consisted of 164 middle school students aged 13–16 (*M* = 14.47, SD = 0.09), of whom 56.7% were girls and 43.3% were boys. The respondents came from families in which 9.1% of mothers and 4.7% of fathers had only primary school education, 13.6% of mothers and 16.8% of fathers had vocational education, 19.5% of mothers and 22.8% of fathers had secondary education, 10.4% of mothers and 15.4% of fathers had a Bachelor’s degree, and 47.4% of mothers and 40.3% of fathers had a Master’s degree or above. Teenagers came from the Pomeranian region of Poland. Their families maintained an average standard of living (*M* Family SES = 6.57; SD = 1.30) as measured by MacArthur’s Scale of Subjective Social Status (Goodman et al., 2001). All measures were administered in the Polish language.

### Procedure and Materials

The study was conducted at the schools the teens’ attended. They were seated in classrooms separately so that they could not communicate with each other and were randomly assigned to one of the eight conditions in two (elevated vs. non-elevated self-esteem) × four (activated single materialism role model – mother, father, peer, or media) experimental design.

#### Elevation of Self-Esteem

The procedure of elevating self-esteem was inspired by a method previously used by Chaplin and John (2007). Teens had to do a word search puzzle and find 10 adjectives (only positive characteristics of people). After that, they had to form 10 sentences about themselves with all of the adjectives they had found (e.g., *I am*… *creative*; *I am*… *ambitious*; *I am*… *friendly*). In the non-elevated condition, teens had to solve a word search puzzle, i.e., 10 nouns referring to everyday life and surroundings, e.g., a cloud, a mountain, a couch. After finding the words, participants had to write them down in a column.

To check the effect of manipulation, the teens in the experimental group were asked to pick one characteristic and describe a situation within the previous 3 months in which they displayed that characteristic and, thus, were satisfied with themselves (“Choose one of the characteristics from the word search puzzle. Recall a situation within the last 3 months when you displayed the characteristic and were satisfied with yourself. Describe the situation”). After the survey, competent judges (psychology experts) (*n* = 4) assessed the content of descriptions of teens in the group with self-esteem manipulation (“Indicate to what extent the content presented in the description indicates that the teenager was proud and satisfied on a scale from 1 – *absolutely not* to 5 – *absolutely yes*”). In assessing the effectiveness of self-esteem manipulation, we took into account the mean and SD of the judges’ ratings and the concordance of the ratings.

#### Activation of the Materialism Role Model

The procedure of activation of materialism role models (i.e., mothers, fathers, peers, and media) was inspired by a method successfully employed in previous studies by Ashikali and Dittmar (2011); Bauer et al. (2012), and Zawadzka et al. (2021). It involved the use of ads, images, and videos with materialistic themes. The activation procedure was divided into two parts. First, in mother, father, and peer conditions, teens answered three identical questions referring to a relevant materialism role model (i.e., “Which of these things would your mother/father/peer choose to make himself/herself happy?”) choosing one out of three answers for each question from separate sets for each condition; the answers were customized to suit the relevant materialism role model, e.g., *their child having a well-paid job in the future* for father/mother or *winning a nationwide inter-school competition* for peers. In the media condition, respondents answered three questions (i.e., “Which of these things do media present as those that bring people happiness?”). The provided answers in all four conditions included materialistic goals only, e.g., *fame, celebritydom, and very high salary, studying at a world-famous/prestigious university*.

Next, respondents in all four conditions were shown visual materials of 36 material goods (e.g., cosmetics, sports shoes, cars, jewelry, backpacks or purses, computers, and luxury vacations) with high price tags and logos of prestigious brands. A preliminary study had confirmed that teens were familiar with the goods/brands used in the visual materials and considered them luxurious. In mother, father, and peer conditions, teens were asked to “indicate at least three things that their mother/father/peer would choose to make herself/himself happy”; the products were customized depending on who the questions referred to. In the media condition, teens indicated “at least three things that would bring the biggest happiness to everybody according to media advertisements and commercials”.

In the neutral condition, teens answered three neutral questions about preferences for colors and places, choosing one out of three suggested neutral responses, e.g., “Which of the following things would you choose to make yourself happy with your job in the future, when you grow up: (a) *working in a room with paintings on the walls*; (b) *working in a room with flowers*; and (c) *working in a room with colorful walls*”; “Which of the following things would you choose to make yourself happy, when you grow up: (a) *more green places in the place where I live*; (b) *more cycle lanes in the place where I live*; and (c) *more playgrounds in the place where I live*.” Next, teens were asked to choose at least three most preferred figures out of 36 colorful figures: i.e., (a) squares, (b) rectangles, (c) ellipses, and (d) triangles (e) polygons.

#### The Measure of the Materialism of Teens

To assess the materialism of teens, we used the AI adapted for teens (AI; Kasser et al., 2014, Study 4). The AI is described in the methodology section of Study 1 above. For this study, the reliability of the REIVO (after recoding items for intrinsic goals) was Cronbach’s α = 0.75 and McDonald’s α = 0.68.

### Results

#### Self-Esteem Manipulation Check

The mean of competent judges’ ratings was *M* = 4,21, SD = 0.53 (Max = 5). Kendall’s W of concordance analysis of competent judges was W = 0.68. Thus, the judges agreed that the descriptions of teens in the experimental group do express pride and self-satisfaction, which means that the manipulation of self-esteem – self-esteem elevation was successful.

#### Self-Esteem, Materialism Role Models, and Materialism of Teens

To check hypothesis 2, regression analyses with categorical variables were performed for each of the tested materialistic models (i.e., mother, father, peer, and media). In accordance with the analysis requirements, the nominal variables (i.e., study conditions) were counted into instrumental variables by binary coding (zero-one) before entering the model. The first variable represented the group primed with both elevated self-esteem and materialism role model (i.e., mother or father or peer or media). The second variable represented the group primed with materialism role model only. The control group was a reference group. The dependent variable was the materialism of teens (REIVO score).

The results showed that only the materialism role model of peers proved significant [*R* = 0.39, *R*^2^ = 0.15, *F*(2,53) = 4.83, *p* = 0.012]. Regression analysis coefficients indicated that the peer condition differed significantly from the control condition on the level of materialism of teens (β = 0.46, *t* = 3.10, *p* = 0.006; LLCI = 0.36, ULCI = 1.56) whereas the self-esteem and peer condition did not differ significantly from the control group (β = 0.21, *t* = 1.43, *p* = 0.11, LLCI = 0.36, ULCI = 1.56). Regression analyses of the tested models for the other conditions were not significant (see Table 2).

**TABLE 2:** Summary of linear regression analysis predicting materialism of adolescent from activation of self-esteem and/or materialism role model (Study 2).

Model	Conditions	B	s.e.	β	t	p	Ba LLCI	Ba ULCI
Peer	SES and MAT	0.46	0.32	0.21	1.43	0.16	–0.07	1.05
	MAT	0.02	0.30	0.46	3.10	0.003	0.358	1.56
		*R* = 0.39, *R^2^* = 0.15, *F*(2,53) = 4.83, *p* = 0.012						
Mother	SES and MAT	–0.10	0.01	0.05	–0.30	0.76	–0.647	0.595
	MAT	0.42	0.02	0.20	1.26	0.21	–0.290	1.10
		*R* = 0.10, *R*^2^ = 0.01, *F*(2,53) = 1.47, *p* = 0.24						
Father	SES and MAT	–0.14	0.31	–0.08	–0.47	0.64	–0.672	0.366
	MAT	0.09	0.31	0.05	0.29	0.77	–535	0.828
		*R* = 0.11, *R*^2^ = 0.01, *F*(2,51) = 0.294, *p* = 0.75						
Media	SES and MAT	–0.04	0.36	–0.02	–0.12	0.91	–0.664	0.640
	MAT	0.41	0.35	0.19	1.18	0.24	–0.215	1.149
		*R* = 0.20, *R* = 0.04, *F*(2,46) = 0.983, *p* = 0.38						

*Linear regression analysis was conducted on the transformed variables; the control group is a reference group. SES and MAT = conditions with both self-esteem and materialism role model activation, MA = conditions with materialism role model activation only; BCa LLCI = bias-corrected accelerated lower 95% CI; BCa ULCI = bias-corrected accelerated upper 95% CI.*

Therefore, hypothesis H2 was confirmed. In other words, Study 2 indicated that elevating self-esteem results in reducing the influence of materialism of peers on the materialism of teens. However, this is not true for the other social role models tested (i.e., mother, father, or media).

## Discussion

The aim of the study was to check whether the self-esteem of teens can help resist the social modeling of materialism. The research conducted so far has focused on the relationship between materialism and self-esteem (Chaplin and John, 2007; Park and John, 2011; Zawadzka and Iwanowska, 2016), on self-esteem as a mediator of the relationship between materialism and parental and peer support (Chaplin and John, 2010), and on self-esteem as a moderator of the relationship between materialism and peer rejection (Jiang et al., 2015). However, the role of self-esteem in resisting materialistic social influences (i.e., mother, father, peer, and media) has not been studied before. Thus, the presented study expands the knowledge on the nature of the relationship between self-esteem and materialism of teens. It is important to note that we conducted both cross-sectional and experimental studies, the latter of which employed various ways of activating self-esteem and materialism role models. The cross-sectional study (1) showed that high self-esteem can help teens resist the influence of materialism on peers, which is not the case for the other materialistic influences tested (i.e., mother, father, and media). The experimental study (2) indicated that elevated self-esteem can decrease peers’ materialism influence on the materialism of teens.

The findings of the first study, that confirm a moderation effect of high self-esteem for peer influence on materialism of teens, may be explained by the fact that this superior strength of this specific influence results from the need to be accepted and the fear of ostracism (Mujiyati and Adiputra, 2018) since peers are particularly important references for the group identity of teens (Erikson, 1968). According to previous studies, low self-esteem is linked to increased susceptibility to peer pressure (Bukowski et al., 2008), while high self-esteem is positively linked to resistance to peer pressure (Chen et al., 2016). In addition, high self-esteem is linked to clear self-beliefs, and teens who have clear self-beliefs are more resistant to social consumption motivation (i.e., imitating peers; Gil et al., 2012).

The absence of a moderation effect for parental models may result from differences between the developmentally based nature of parental influence and that of the peers. In teens, parental influence is diminished in favor of peer influence (Maccoby, 1992; Schonpflug, 2001). Parental influence refers to intergenerational transmission based on the emotional bond (Tucker and Updegraff, 2009), while peer influence relates to a relationship in which teens can choose the people they will form close ties with (Furman and Buhrmester, 1992). The influence of parents, who are the first agents of socialization, affects the hierarchy of teens’ values, and peer influence affects preferences concerning everyday behaviors (e.g., what music to listen to, what to wear, where to hang out; Maccoby, 1992; Schonpflug, 2001; Richins, 2017). Thus, self-esteem may not moderate the relationship between the influence of materialism of parents and materialism of teens.

The absence of a moderation effect in the case of media may be due to the fact that the relationship between the media and advertisement exposure and teens’ materialism is weaker than the relationship between other materialistic influences and teens’ materialism. Previous studies also show that the relationship between materialistic media and teens’ materialism does occur but is rather weak (cf. Buijzen and Valkenburg, 2003; Opree et al., 2014). Adopting previously used measures, we examined the frequency of teen exposure to media (Schor, 2004). However, a growing number of studies on adults indicate that there are other variables that should be analyzed when measuring the influence of media, such as active processing during viewing (Shrum et al., 2005), commercial portrayals of characters (Richins, 1987), the purpose of using media (Richins, 2017), or active participation in social media and social networks (Noar and Harrington, 2012).

It is worth mentioning in this study that Study 1 did not confirm the conclusions from the research of predecessors on American teens that indicate a significant negative relationship between self-esteem and materialism of teens (Chaplin and John, 2007, 2010; Park and John, 2011). As stated in the text, there are studies showing that the relationship between self-esteem and materialism depends on how teens define themselves (Zhang and Hawk, 2019) and that the relationship between self-esteem and materialism may also depend on one’s origin or culture (see Zawadzka et al., 2020). Thus, it can be assumed that the negative relationship between self-esteem and materialism may be related to the culture in which the teen respondents have been growing up.

Our experimental study also confirmed that elevating the self-esteem of teens reduces the impact of the materialism of peers but not that of the other materialistic models. In this respect, the experimental study results echo the results of the cross-sectional one. Based on these results, it can be assumed that peers’ materialistic influence is less stable than that of parents and can be changed by boosting the teen’s self-esteem. Boosting self-esteem is not related to lessening the materialistic influence of parents because parental influence is more stable since parents are the first social models and self-esteem is largely determined by the relationship with parents and the upbringing of the child in the period prior to adolescence (cf. Coopersmith, 1967; Harter, 1983).

### Limitations

The presented research also has its limitations. First, following our predecessors the measurement of media materialism used in the cross-sectional study (1) focuses on media use frequency. Considering the conclusions from studies on adults, it would be a good idea to extend the next study to include additional variables such as active processing during viewing or materialistic purposes of using media (cf. Richins, 1987; Shrum et al., 2005), which may be of importance for materialistic aspirations. Second, the studies focus on self-esteem that is defined as a positive attitude and a good opinion of oneself. However, as known from previous research, the relationship between materialism and self-esteem may be related to how people define themselves (cf. Zhang and Hawk, 2019) and in which areas of the self they build their self-worth contingencies (cf. Nagpaul and Pang, 2017; Chen et al., 2020). Taking account of the fact that activating intrinsic contingencies of self-worth and extrinsic contingencies of self-worth are linked to materialism in different ways (cf. Nagpaul and Pang, 2017), further studies should extend the analysis of self-esteem as a mediator of materialistic influences, such as the topic of self-worth contingencies. Third, the teens came from families with average family SES. Previous research suggests that the level of family SES (low and high) may be related to the self-esteem of teens and their materialism (cf. Nairn and Opree, 2021). Thus, subsequent research should be expanded to include the analysis of family SES as a mediator of self-esteem moderation in the relation between the materialism of teens and that of their peers.

Despite the limitations indicated above, the research presented here clearly demonstrated that self-esteem may allow predicting the effect of materialistic peer influences on materialism if teens and that elevating self-esteem can work as a resource helping teens, i.e., 13–16-year olds from families with average SES resist the materialistic influence of peers.

### Practical Implications

Conclusions from the research on the role and function of self-esteem in healthy functioning carried out so far are not obvious. In the present study, it was shown that good self-esteem can be an effective way to be less prone to being influenced by peers, for example, overpaying attention to materialistic goals that have negative consequences for health (cf. Kasser and Ryan, 1993; Cohen and Cohen, 1996; Williams et al., 2000; Twenge et al., 2010; Manolis and Roberts, 2012; Kasser et al., 2014; Tsang et al., 2014; Moldes and Ku, 2020). Some researchers uphold the claim that self-esteem is essential for the functioning and allows predicting the effects of actions and even life success (Donnellan et al., 2005; Schimel et al., 2008), while others point to its limited value or even the burden that high self-esteem may put on individuals (Baumeister et al., 1996, 2003; Heatherton and Vohs, 2000). The presented studies also have practical implications. They suggest that a way to make teens resistant to the omnipresent materialistic social influences is to undertake activities aimed at strengthening their self-esteem, especially in the context of peer connections.

Thus, the obtained results may contribute to the development of intervention programs promoting boosting self-esteem on both cognitive (perception of self-worth) and social (perception of oneself in relationships with others) levels (Harter, 1999) to make teens more resistant to the influence of peer role models on materialism. The conducted experiment shows that effective strengthening of self-esteem on the cognitive level may include exercises such as “best possible self,” a classic of positive psychology (King, 2001; Sheldon and Lyubomirsky, 2006) or “strengths exploration” (Seligman et al., 2005). The results of the correlational study show that intervention programs, such as measures to strengthen the self-esteem of teens and a sense of support in the peer group, may be beneficial for the health of teens (cf. Harter, 1999; Morris, 2009).

Contrary to previous studies on self-esteem interventions that did not indicate that self-esteem interventions can enhance the wellbeing of the individual (Guindon, 2010), the results obtained in the current studies do indicate that self-esteem may be a resource reducing the impact of peer materialism, which carries negative effects on the wellbeing of teens.

## Data Availability Statement

The raw data supporting the conclusions of this article will be made available by the authors, without undue reservation.

## Ethics Statement

The studies involving human participants were reviewed and approved by Ethics Committee Department of Psychology, University of Gdańsk. Written informed consent to participate in this study was provided by the participants’ legal guardian/next of kin.

## Author Contributions

AZ: project administration, supervision, conceptualization, methodology, formal analysis, and writing the original draft. JB: methodology, investgation, and writing the original draft and data curation. MI and AL-W: methodology, investigation, and writing the original draft. All authors read and approved the manuscript.

## Conflict of Interest

The authors declare that the research was conducted in the absence of any commercial or financial relationships that could be construed as a potential conflict of interest.

## Publisher’s Note

All claims expressed in this article are solely those of the authors and do not necessarily represent those of their affiliated organizations, or those of the publisher, the editors and the reviewers. Any product that may be evaluated in this article, or claim that may be made by its manufacturer, is not guaranteed or endorsed by the publisher.

## Footnotes

1 This study is a part of bigger research on Social determinants of materialism in teenagers.
